# Dynamics of Auxin and Cytokinin Metabolism during Early Root and Hypocotyl Growth in *Theobroma cacao*

**DOI:** 10.3390/plants10050967

**Published:** 2021-05-12

**Authors:** Alexandre Mboene Noah, Rubén Casanova-Sáez, Rolande Eugenie Makondy Ango, Ioanna Antoniadi, Michal Karady, Ondřej Novák, Nicolas Niemenak, Karin Ljung

**Affiliations:** 1 Department of Biochemistry, Faculty of Science, University of Douala, Douala P.O. Box 24157, Cameroon; 2 Umeå Plant Science Centre, Department of Forest Genetics and Plant Physiology, Swedish University of Agricultural Sciences (SLU), 90183 Umeå, Sweden; ruben.casanova.saez@slu.se (R.C.-S.); ioanna.antoniadi@slu.se (I.A.); michal.karady@seznam.cz (M.K.); ondrej.novak@upol.cz (O.N.); Karin.Ljung@slu.se (K.L.); 3 Laboratory of Plant Physiology and Biochemistry, Department of Biological Science, Higher Teachers’ Training College, University of Yaounde I, Yaounde P.O. Box 47, Cameroon; rolandemicky@yahoo.fr (R.E.M.A.); niemenak@yahoo.com (N.N.); 4 Laboratory of Growth Regulators, Faculty of Science, Institute of Experimental Botany of the Czech Academy of Sciences, Palacký University, Šlechtitelů 27, CZ-78371 Olomouc, Czech Republic

**Keywords:** *Theobroma* *cacao*, auxin, cytokinin, phytohormone metabolism, root development

## Abstract

The spatial location and timing of plant developmental events are largely regulated by the well balanced effects of auxin and cytokinin phytohormone interplay. Together with transport, localized metabolism regulates the concentration gradients of their bioactive forms, ultimately eliciting growth responses. In order to explore the dynamics of auxin and cytokinin metabolism during early seedling growth in *Theobroma cacao* (cacao), we have performed auxin and cytokinin metabolite profiling in hypocotyls and root developmental sections at different times by using ultra-high-performance liquid chromatography-electrospray tandem mass spectrometry (UHPLC-MS/MS). Our work provides quantitative characterization of auxin and cytokinin metabolites throughout early root and hypocotyl development and identifies common and distinctive features of auxin and cytokinin metabolism during cacao seedling development.

## 1. Introduction

*Theobroma cacao* (cacao) is an economically important crop whose beans are the primary ingredient of chocolate. The cacao value chain involves farmers, commercial intermediates, manufacturing industries, and consumers [1]. Cacao derivatives are used in confectionery, as well as in cosmetic and pharmaceutical industries, while chocolate consumption is so deeply integrated into human habits that it has become a cultural commodity. The increased demand for cacao necessitates extensive research on the cacao tree developmental biology. Seeds promote geographical dispersion of genetic diversity. Cacao seeds germinate immediately after completing their maturation and they do not survive drying during ex situ conservation. The emergence of the radicle from the seed coat marks the transition from germination to the seedling establishment phase. Seedling establishment is a vulnerable process during which the plant grows heterotrophically until the first leaves emerge, and the seedling acquires autotrophic capacity. Therefore, this phase is critical for plant survival and a determinant of yield in agricultural crops. The developmental window of seedling establishment in *T. cacao* can be centered on the root differentiation and hypocotyl elongation occurring between 4 and 10 days after germination [2]

The plant root system consists of the primary root formed during embryogenesis and lateral roots that are formed postembryonically from the primary root [3]. Along the proximal-distal axis, starting from the root tip and moving upwards to the root-hypocotyl junction, the primary root can be divided into three main developmental zones which comprise the meristematic zone (Mz), the elongation zone (Ez), and the differentiation zone (Dz). The Mz is mainly composed of mitotically active cells from which all root tissues originate. The Ez originates at the basal meristem, also referred to as the transition zone, where division rates slow and cells begin to increase in size [4,5]. Root cells progressively undergo cell elongation along the Ez, thus allowing root growth. In the Dz, cells complete their differentiation programs according to the tissue identity. A classical hallmark of the root Dz is the appearance of epidermal root hairs and the initiation and emergence of lateral roots [6]. Lateral root formation and patterning take place across all the different developmental zones of the primary root. Priming and prebranching (specification of cells that will initiate a lateral root), are spatially restricted to the lateral root cap and the oscillation zone, a region that encompasses the basal meristem and the Ez [7,8,9]. Later developmental stages in lateral root formation such as initiation, primordium organogenesis, and emergence take place in the Dz [6].

Primary and lateral root developmental stages are tightly regulated by a complex interplay of different plant hormones, particularly auxin and cytokinins (CKs) [10,11,12]. Auxin–cytokinin interactions are crucial for the positioning of the transition zone, which ultimately defines root meristem size and primary root growth [13]. Lateral root patterning and development along the primary root highly depend on periodic auxin pulses derived from the lateral root cap and on an increased auxin response capacity of subsets of pericycle cells [14]. Cytokinin acts as an endogenous inhibitor of lateral root initiation and counteracts the stimulatory effect of auxin on cell division [15,16,17]. Hypocotyl growth depends on auxin transport and metabolism and light perception by photoreceptors [18,19,20]. In contrast to roots, auxin accumulation in hypocotyls triggers cell elongation and growth [21]. Cytokinins act as differential modulators of hypocotyl cell elongation depending on light [22,23].

Because auxin and cytokinin-mediated responses depend upon their concentration gradients and interaction with specific receptors, precise characterization of the levels of these hormones and their metabolites help in clarifying their role in plant growth and development. The main auxin in plants, indole-3-acetic acid (IAA), is primarily synthesized from tryptophan via the formation of indole-3-pyruvic acid [24]. Reversible and irreversible inactivation of IAA operate to regulate IAA gradients during plant development. IAA conjugates with specific amino acids and glucose are well-known reversible storage forms [25]. The oxidized form of IAA, 2-oxindole-3-acetic acid (oxIAA), and the IAA conjugates to aspartate (IAAsp) and glutamate (IAGlu) are irreversible catabolites that regulate auxin levels after accumulation [26,27,28,29] or during regulated auxin minima formation [13,20]. IAA, oxIAA, IAAsp and IAGlu are thus key indicators of the auxin metabolic status in plant tissues. Cytokinins (CKs) are adenine-derived compounds that are grouped into aromatic or isoprenoid CKs, depending on the side chain of the adenine. Isoprenoid CKs are more common in plants and their free-base forms isopentenyladenine (iP), trans-zeatin (tZ), cis-zeatin (cZ), and dihydrozeatin (DHZ), are the bioactive hormones [30,31]. Biosynthesis of CKs initiates from the tRNA degradation or the isopentylation of adenine nucleotides and proceeds by sequential hydrolysis of the phosphates and the ribose of the CK nucleotides and nucleosides, respectively, or directly by phosphoribohydrolysis of CK nucleotides [32,33]. CK nucleotides and ribosides are thus precursors of the active forms. CKs are irreversibly inactivated by CKX-mediated cleavage of the isoprenoid side chain in iP-, tZ- and cZ-types and reversibly inactivated by glycosylation on the hydroxyl group of tZ- and cZ-side chain, giving rise to *O*-glucosides [32,34]. Glycosylation of the CK purine ring at positions 3, 7, or 9 originates N-glucosides, which serve as catabolites or storage forms depending on the species and the CK type [35,36].

Quantitative changes in auxin and cytokinin and their metabolites in different plant tissues have been reported in several species but mostly in Arabidopsis [15,37,38,39,40,41,42], rice [43], maize [44], Populus [45,46], bryophytes [47], and algae [48]. As CK and auxin are closely interconnected and indispensable for plant growth and response to stresses, examining their fluxes and levels during development is very important, especially in agriculturally important crops. Dynamic changes in auxin and CK metabolism during *Theobroma cacao* (cacao) development remain largely unexplored. In order to fill this gap, we have investigated the endogenous concentrations of auxin and cytokinin metabolites in cacao roots and hypocotyls, at different developmental stages and in different root developmental zones. Concentration gradients over the course of development as well as particularities of auxin and cytokinin metabolism in cacao are reported and discussed.

## 2. Results

**Figure 1:**
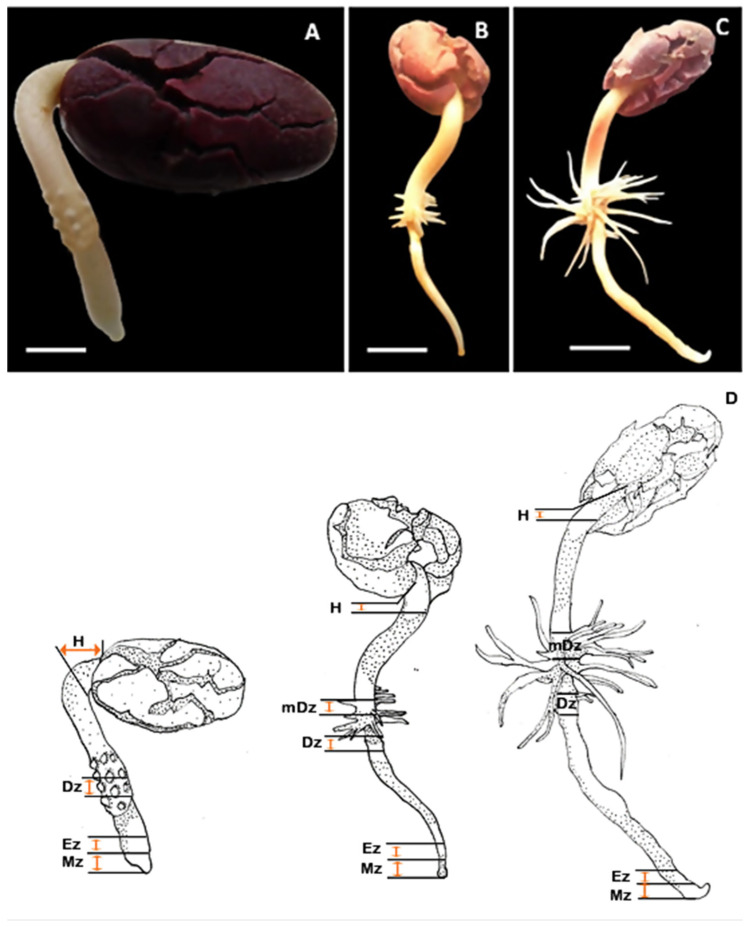
Developmental stages of the cacao root system analyzed in this study. Representative image of cacao seedlings at (**A**) 4, (**B**) 7, and (**C**) 10 DAI (days after initiation of germination). 4, 7, and 10 DAI correspond to BBCH07, BBCH08, and BBCH10 stages, respectively, according to the BBCH (Biologische Bundesantalt, Bundessortenamt and CHemische Industrie) scale codification (Niemenak et al., 2010) [2]. Scale bar represents (**A**) 1 cm and (**B**,**C**) 0.5 cm. (**D**) Illustration of the cacao tissues sampled in this study. Mz, meristematic; Ez, elongation zone; Dz, differentiation zone; mDz, mature differentiation zone; H = hypocotyl segment.

To explore the dynamics of auxin and cytokinin metabolism during early seedling development in T. cacao, endogenous concentrations of auxin and cytokinin metabolites were quantified in cacao root and hypocotyl sections. Hypocotyl (H) and four sections of the primary root including meristematic zone (Mz), elongation zone (Ez), differentiation zone (Dz) and the mature differentiation zone (mDz) were sampled (Figure 1). Correspondence between the developmental stages sampled in this study and the T. cacao BBCH (Biologische Bundesantalt, Bundessortenamt and CHemische Industrie) scale codification described by Niemenak et al., 2010 [2] was established. The tissue sections were collected at three different times: 4, 7, and 10 days after initiation of germination (DAI). This timeframe recapitulated significant root and hypocotyl developmental events during early seedling growth in cacao (Figure 1). At 4 DAI (BBCH07), elongation of the radicle had typically produced a primary root up to 1 cm long bearing protrusions at the upper region (Figure 1A). The mDz was not yet formed at 4 DAI. Visible lateral root outgrowths were apparent at 7 DAI (BBCH08, Figure 1B). By 10 DAI (BBCH10) the lateral root system became denser and longer, while the primary root continued to grow (Figure 1C). Other significant morphogenic changes noticeable in our experimental conditions include an apical hook, visible at 4 DAI (BBCH07), and a remarkable hypocotyl elongation after hook opening during the transition from 4 to 7 DAI. The cotyledons remained closed over the experimental timeframe.

### 2.1. Auxin Metabolite Profiling in Developing Cacao Root and Hypocotyl

**Figure 2:**
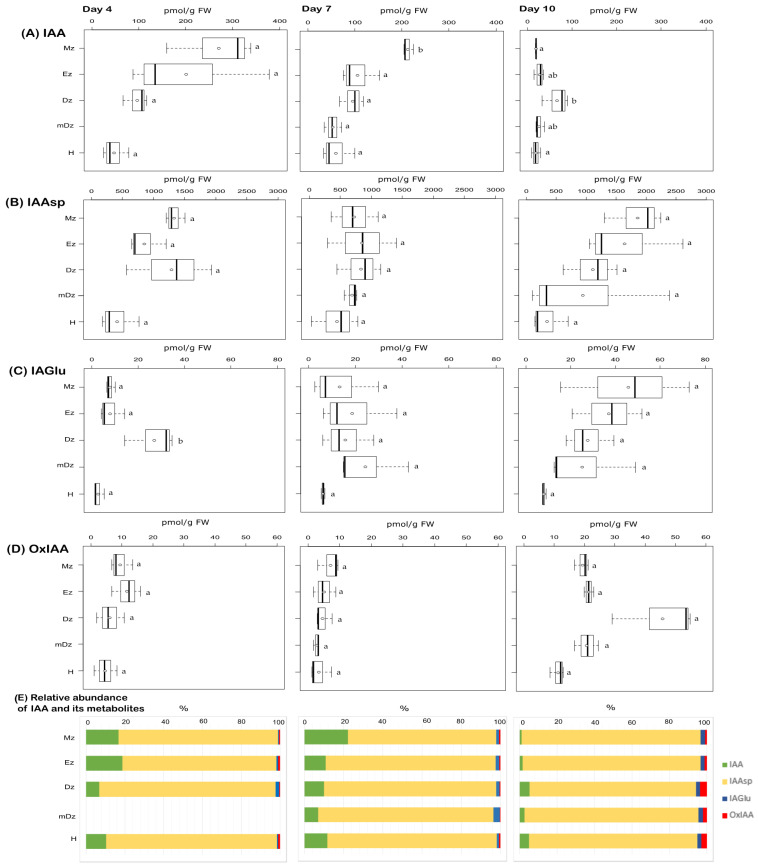
Quantification of auxin metabolites in hypocotyl and developmental root sections of cacao seedlings. Levels of (**A**) indole-3-acetic acid (IAA), (**B**) indole-3-acetyl-L-aspartic acid (IAAsp), (**C**) indole-3-acetyl-L-glutamic acid (IAGlu), and (**D**) 2-oxindole-3-acetic acid (oxIAA) were determined from the samples described in Figure 1. The dark vertical line indicates median; boxes represent 25th and 75th percentiles; horizontal lines represent maximum and minimum values; circles indicate mean values. Significant variations in concentration determined by Welch’s test post hoc, *p* ≤ 0.05 among hypocotyl and primary root sections are indicated by letters, *n* = 3 independent biological replicates; FW = fresh weight. (**E**) Percent proportion of each auxin metabolite.

To gain insight into the auxin metabolic status during cacao root and hypocotyl development, endogenous concentrations of the free/active IAA, the conjugates IAAsp, IAGlu, and the oxidative catabolite oxIAA (Supplementary Table S1) were quantified from the collected samples (Figure 2 and Supplementary Table S2). At 4 and 7 DAI, IAA levels were found higher at the Mz compared to other root developmental zones and the hypocotyl; this difference was significant only at 7 DAI (Figure 2A). A redistribution of IAA across root developmental zones was noticed at 10 DAI, when IAA preferentially accumulated within the Dz segment at a level representing about 48% of total IAA from the root system (Figure 2A). In 10-day-old seedlings, a sharp reduction in IAA concentration was observed in all segments, except for the Dz root segment, where IAA content was quite stable throughout the time points (Figure 2A).

Levels of the IAA conjugates IAAsp and IAGlu were comparable across all segments and days analyzed, with the exception of a peak of IAGlu levels around the Dz at 4 DAI (Figure 2B,C). The catabolite oxIAA was found at similar levels across root segments and hypocotyls from 4- and 7-day-old seedlings (Figure 2D). At 10 DAI, oxIAA levels were overall increased in all root segments and hypocotyls, with the highest concentration found in the Dz (Figure 2D). Worth highlighting is that oxIAA was a minor IAA catabolite compared to the conjugates IAAsp and IAGlu across our developmental segments and time points (Figure 2E), contrary to what has been traditionally observed in many other plant species [27,40,45,47,48]. This suggests that IAA conjugation to Asp and Glu is a major catabolic pathway regulating endogenous IAA levels in roots and hypocotyl during early cacao seedling development.

### 2.2. Cytokinin Metabolite Profiling in Developing Cacao Root and Hypocotyl

In order to explore the dynamics of cytokinin metabolism during cacao root and hypocotyl development, the levels of twenty-six isoprenoid cytokinins were determined. Twelve of these cytokinin forms were detected from the collected samples under our experimental conditions (Supplementary Tables S1 and S3). These included three active cytokinin free bases (iP, tZ and cZ) and some of their corresponding nucleotides, ribosides, and glucosyl-conjugates. Specifically, the measured cytokinin pool was composed of four iP-type cytokinins, five tZ-type cytokinins, one DHZ-type cytokinins, and two cZ-type cytokinins (Supplementary Table S1).

**Figure 3:**
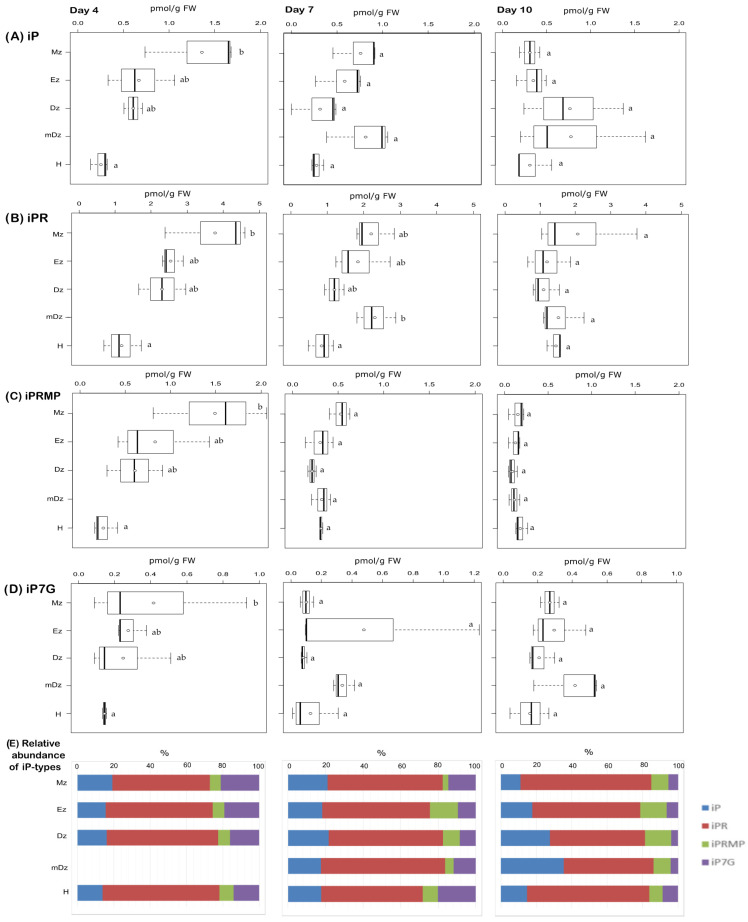
Quantification of iP-type cytokinins in hypocotyl and developmental root sections of cacao seedlings. Levels of (**A**) isopentenyladenine (iP), (**B**) isopentenyladenosine (iPR), (**C**) isopentenyladenosine-5′-monophosphate (iPRMP), and (**D**) isopentenyladenine-7-glucoside (iP7G) were determined from the samples described in Figure 1. Isopentenyladenine-9-glucoside (iP9G) levels were below the limit of detection in all samples. Dark vertical line indicates median; boxes represent 25th and 75th percentiles; horizontal lines represent maximum and minimum values; circles indicate mean values. Significant variations in concentration determined by Welch’s test post hoc, *p* ≤ 0.05 among hypocotyl and primary root sections are indicated by letters, *n* = 3 independent biological replicates; FW = fresh weight. (**E**) Percent proportion of each iP-type cytokinin metabolite.

At 4 DAI, levels of the free-base iP peaked at the Mz and decreased towards the H; this concentration gradient was not maintained in later stages (Figure 3A). The iP precursors iPR and iPRMP also showed a similar concentration gradient, especially at 4 DAI (Figure 3B,C). In hypocotyls, iP levels remained steady over the time points but usually lower than in roots (Figure 3A). Levels of the CK catabolite iP7G, which was the only CK N-glucoside detected under our conditions, showed a tissue gradient similar to iP at 4 DAI, while their levels were comparable across tissue segments at 7 and 10 DAI (Figure 3D).

At 4 DAI, levels of the free-base iP peaked at the Mz and decreased towards the H; this concentration gradient was not maintained in later stages (Figure 3A). The iP precursors iPR and iPRMP also showed a similar concentration gradient, especially at 4 DAI (Figure 3B,C). In hypocotyls, iP levels remained steady over the time points but usually lower than in roots (Figure 3A). Levels of the CK catabolite iP7G, which was the only CK N-glucoside detected under our conditions, showed a tissue gradient similar to iP at 4 DAI, while their levels were comparable across tissue segments at 7 and 10 DAI (Figure 3D).

**Figure 4:**
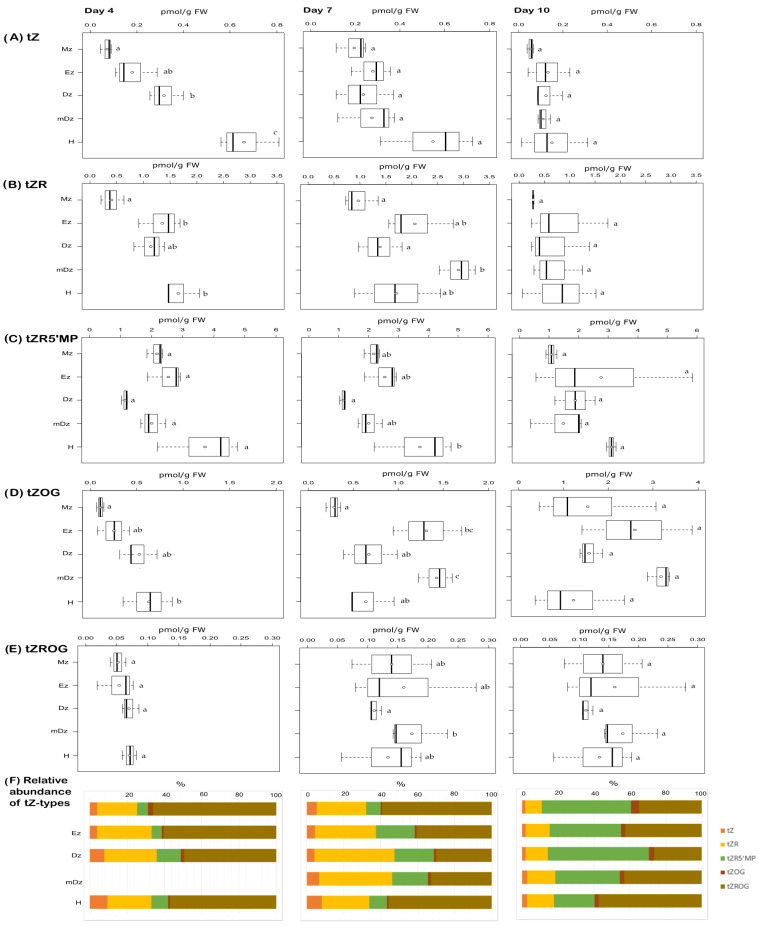
Quantification of tZ-type cytokinins in hypocotyl and developmental root sections of cacao seedlings. Levels of (**A**) trans-zeatin (tZ), (**B**) trans-zeatin riboside (tZR), (**C**) trans-zeatin riboside-5′-monophosphate (tZR5′MP), (**D**) trans-zeatin-O-glucoside (tZOG), and (**E**) trans-zeatin riboside-*O*-glucoside (tZROG) were determined from the samples described in Figure 1. The levels of trans-zeatin-7-glucoside (tZ7G) and trans-zeatin-9-glucoside (tZ9G) were below the limit of detection in all samples. The dark vertical line indicates median; boxes represent 25th and 75th percentiles; horizontal lines represent maximum and minimum values; circles indicate mean values. Significant variations in concentration determined by Welch’s test post hoc, *p* ≤ 0.05 among hypocotyl and primary root sections are indicated by letters, *n* = 3 independent biological replicates; FW = fresh weight. (**F**) Percent proportion of each tZ-type cytokinin metabolite.

In contrast to iP, the levels of tZ were higher at the hypocotyls while decreasing towards the Mz at 4 and 7 DAI (Figure 4A). At 10 DAI tZ concentrations were similar across the root and in the hypocotyl. Similar to tZ, the levels of the tZ precursors tZR and tZRMP were usually higher at the hypocotyl at 4 and 7 DAI but not later (Figure 4B,C). The tZ storage form tZOG and tZROG became more abundant in all sections as the seedlings developed (Figure 4D,E).

**Figure 5:**
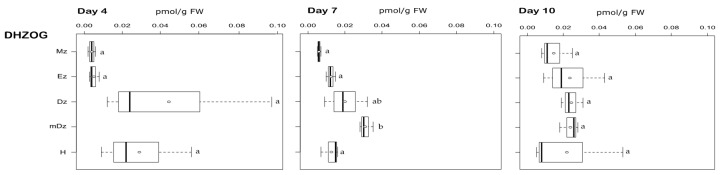
Quantification of DHZ-type cytokinins in hypocotyl and developmental root sections of cacao seedlings. Levels of dihydrozeatin-*O*-glucoside (DHZOG) were determined from the samples described in Figure 1. The levels of dihydrozeatin (DHZ), dihydrozeatin riboside (DHZR), dihydrozeatin riboside-5′-monophosphate (DHZRMP), dihydrozeatin riboside-*O*-glucoside (DHZROG), dihydrozeatin-7-glucoside (DHZ7G), and dihydrozeatin-9-glucoside (DHZ9G) were below the limit of detection in all samples. The dark vertical line indicates median; boxes represent 25th and 75th percentiles; horizontal lines represent maximum and minimum values; circles indicate mean values. Significant variations in concentration determined by Welch’s test post hoc, *p* ≤ 0.05 among hypocotyl and primary root sections are indicated by letters, *n* = 3 independent biological replicates; FW = fresh weight.

Among DHZ-types, only the storage form DHZOG was detected. It was present at very low concentrations, and their levels were similar across the root and in hypocotyls over the time course of the experiment, with a noticeable concentration peak in the mDz at 7 DAI (Figure 5).

**Figure 6:**
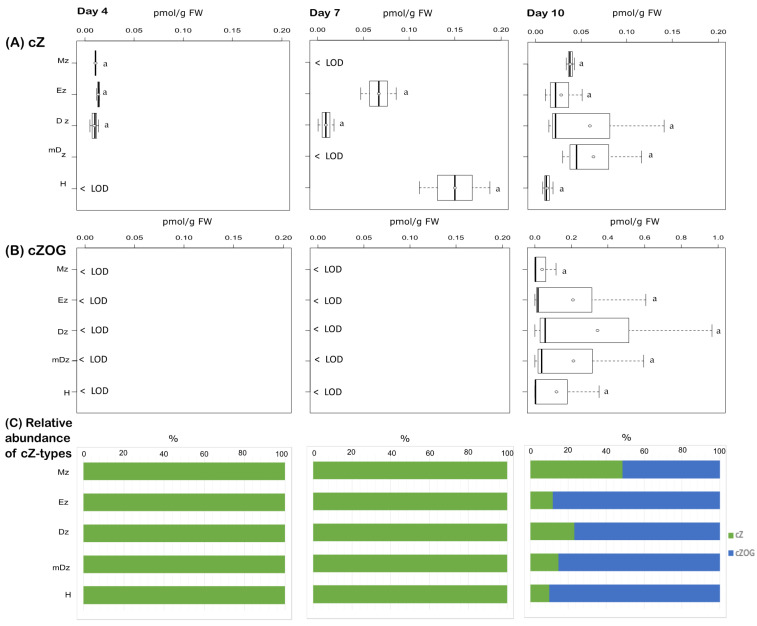
Quantification of cZ-type cytokinins in hypocotyl and developmental root sections of cacao seedlings. Levels of (**A**) cis-zeatin (cZ) and (**B**) cis-zeatin-*O*-glucoside (cZOG) were determined from the samples described in Figure 1. The levels of cis-zeatin riboside (cZR), cis-zeatin riboside-*O*-glucoside (cZROG), cis-zeatin riboside-5′-monophosphate (cZR5’MP), cis-zeatin-7-glucoside (cZ7G), and cis-zeatin-9-glucoside (cZ9G) were below the limit of detection in all samples. The dark vertical line indicates median; boxes represent 25th and 75th percentiles; horizontal lines represent maximum and minimum values; circles indicate mean values. Significant variations in concentration determined by Welch’s test post hoc, *p* ≤ 0.05 among hypocotyl and primary root sections are indicated by letters, *n* = 3 independent biological replicates; FW = fresh weight. (**C**) Percent proportion of each cZ-type cytokinin metabolite.

We also determined the levels of cZ and found that this CK base was present at very low levels, below the limit of detection of our method in many cases, and no statistical difference was observed across all tissues sampled (Figure 6A). Additionally, we were able to detect the cZ storage form cZOG in hypocotyls and roots at 10 DAI but not at earlier stages (Figure 6B).

**Figure 7:**
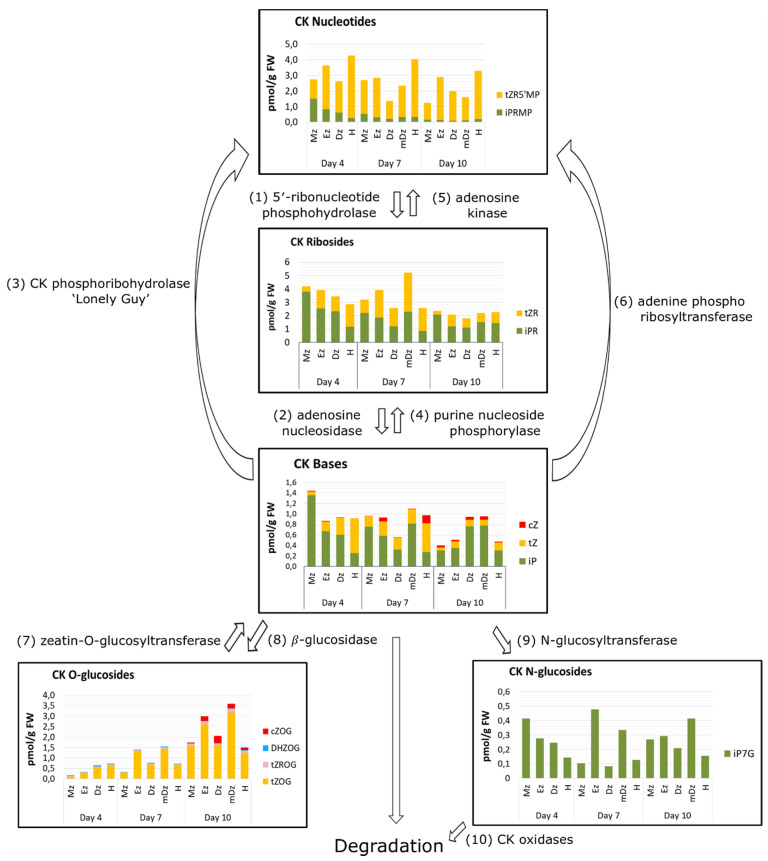
Overview of the cytokinin metabolism in developing cacao seedlings. Results of total CKs are mean values ± SD, *n* = 3 independent biological replicates. The metabolic pathways of different CKs groups are adapted from Antoniadi et al. (2015) [37]. Numbers in parentheses indicate CK biosynthetic and degrading enzymes.

The cytokinin content within developing seedling tissues can also be considered according to their functional types: the bioactive forms (free CK bases), the biosynthetic precursors (CK nucleotides and CK ribosides); the cytokinin storage forms (CK *O*-glucosides), and the cytokinin degradation forms (CK N-glucosides). In general, iP was the most abundant CK base in root developmental sections across our time course, while tZ was the major CK in developing hypocotyls at 4 and 7 DAI (Figure 7). The precursor iPR was also a major CK riboside in roots at 4 DAI and in roots and hypocotyls at 10 DAI, while tZR was more abundant in hypocotyls at 4 DAI and in roots and hypocotyls at 10 DAI. tZRMP and tZOG were the most abundant CK nucleotides and *O*-glucosides, respectively, across all sections and time points (Figure 7).

## 3. Discussion

**Figure 8:**
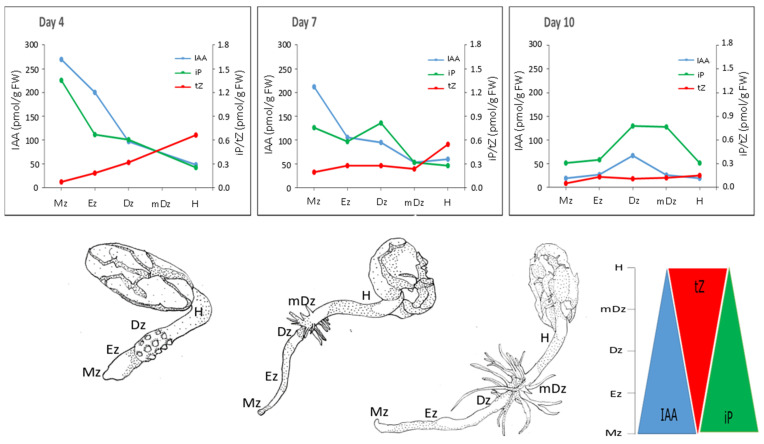
Summary of the dynamic changes in the contents of IAA, tZ, and iP in root developmental zones and hypocotyls during early cacao seedling growth (from 4, 7, and 10 DAI).

The levels of IAA are spatiotemporally regulated within plant tissues in order to generate asymmetric gradients, which confer key positional information to cells and thus modulate developmental zonation [7,49,50]. Polar auxin transport and, particularly, a feedback loop between IAA and its transporters are postulated to be determinant for establishing and maintaining IAA gradients [51,52,53,54,55]. Together with transport, local IAA biosynthesis [56] and degradation [20,57] are required for auxin homeostasis and gradient formation during organ development. This study revealed an IAA concentration maximum at the root Mz and decreasing levels towards the Dz (Figure 8), which is in accordance with the well-characterized IAA gradient in the root from the model plant Arabidopsis [41,58]. The transition from day 7 to day 10 was marked by a substantial general decrease of IAA concentration in the root tissues investigated, especially in the Mz and Ez (Figure 2A). This decrease in IAA levels at 10 DAI was less prominent at the Dz, which resulted in the loss of the IAA maximum at the Mz and the establishment of the Dz as the root developmental zone with the highest IAA concentration (Figure 2A).

While the general decrease in IAA levels at 10 DAI might be related to a dilution of the IAA concentration as root cells grow [59], the loss of the IAA maximum at the Mz in cacao is an unexpected observation, as such maximum is required for sustaining and organizing root growth [41,58]. Studies on Arabidopsis early seedling development showed that lateral root emergence is highly dependent on IAA produced in young leaves and transported to the root, while lateral root initiation requires high IAA levels at the primary root tip [60,61]. Around day 10 after germination, the Arabidopsis root gains the capacity of synthesizing IAA and lateral root emergence becomes less dependent on IAA transport from the aerial tissues [62]. Because the cotyledons were not expanded and the leaves not yet developed in cacao seedlings 10 DAI (BBCH10) under our experimental conditions (Figure 1), the lack of IAA supply from developing aerial tissues might then contribute to the drop in root IAA levels at 10 DAI (Figure 2A). Further investigation and additional corroboration are, nevertheless, needed to clarify such IAA redistribution in developing cacao roots observed in this work.

Irreversible inactivation of IAA in plants mainly occurs through the formation of the IAA-amino acid conjugates IAAsp and IAGlu [29,63], and the oxidation of IAA to oxIAA [27,28,62]. In Arabidopsis, oxIAA is considered the major catabolite regulating IAA levels, because (i) their endogenous levels are about two orders of magnitude higher than those of IAAsp and IAGlu [28,40] and (ii) oxIAA levels increase promptly and to a much higher extent than those of the conjugates [26,27]. The current study revealed that the IAA amino acid conjugates IAAsp and IAGlu were more abundant than oxIAA along the root and hypocotyl throughout the time course of the experiment (Figure 3). Particularly, IAAsp was found at very high concentrations, indicating that conjugation to IAAsp, rather than IAA oxidation, is a major pathway regulating IAA levels during early seedling development in cacao. Such a particular prevalence of conjugation over oxidation for metabolic inactivation of IAA has been very recently reported in conifers [64] and in the lycophyte *Selaginella moellendorffii* [65]. The establishment of IAA oxidation as the major route for auxin catabolism is proposed to have occurred with the appearance of angiosperms [65,66,67]. This study first evidences the prevalence of conjugation over oxidation for IAA metabolic inactivation in an angiosperm.

Cytokinins regulate many growth and developmental processes throughout the plant’s life cycle, primarily due to their role in controlling cell division and differentiation [68]. In growing seedlings, CKs modulate lateral root organogenesis [15,16], root meristem size [13], and elongation of the hypocotyl [23], among other processes. Levels of the 4 active CK free bases and their metabolites have been quantified in different tissues from different plant species [38,44,45,46,69,70]. These studies have found the four CK active bases to be differentially abundant in different plant tissues, suggesting that each CK type might play specific roles. However, it might just be related to the differential bioactivity of the active forms. In general, tZ and iP are considered as the most active CKs, while cZ presents lower biological activity [69,71].

In our study, we have performed quantification of twenty-seven isoprenoid CK metabolites in cacao. Twelve CKs including bioactive, transport, and storage forms as well as cytokinin precursors were detected from developing cacao seedlings during the time course of our experiments (4-10 DAI).

The CK free bases iP, tZ, DHZ, and cZ, which are the biologically active forms [28] can be found differentially abundant in different tissues and plant species. In Arabidopsis root sections, representing lateral root development sites, cZ was the most abundant active cytokinin [15] while in the apical 1 mm of primary root iP was the predominant active CK [70]. In root of cacao young seedlings, iP and tZ were the prevailing active CKs (Figure 7), as was also described in pea [69]. Similarly, tZ and cZ were the most abundant CK active forms in roots of rice [72]; however, iP was found the dominant active CK in *Lotus japonicus* roots [73].

Gradients of the active CK exists in plant tissues and can be relevant for plant growth and development [37,45,74]. In cacao, both iP and tZ followed concentrations gradients across root developmental sections and hypocotyls, with iP—in parallel with IAA concentrations—being more abundant in the root Mz, and tZ more abundant in the hypocotyl (Figure 8). iP was the only active cytokinin that was specifically abundant in the root apex, which is similar to Arabidopsis [70]. In accordance, Arabidopsis seedlings expressing TCSn:GFP, a synthetic promoter fusion with green fluorescent protein reporting global cytokinin response in vivo, presented a spatial maximum of cytokinin response at the Mz of the root (around stele initials) following iP [75].

cZ was present at very low levels in cacao roots and hypocotyls, while most of their metabolites were below the limit of detection. The fact that cZOG was detectable from 10 DAI might indicate that cZ metabolism play more relevant roles later in seedling development. This would not be surprising as the group of cZ-type CKs has been shown the predominant CKs in specific tissues, environmental conditions, and developmental stages in several plant species [69]. For example, cZ (along with tZ) was found the most prevalent active CK in Arabidopsis and rice roots [42,72]. In maize, cZ along with iP are the most abundant active CKs [44]. However, in pea root segments, DHZ and cZ compounds were at hardly detectable levels [74], similar to what we observed in cacao.

CK nucleotides are thought to play a central role in the regulation of cytokinin levels as they are readily converted to both the ribosides precursors and/or directly to the active free base forms [33]. Cytokinin N-glucosides are considered terminal products of the irreversible active cytokinin deactivation and thus are part of a detoxification pathway for cytokinins [76], although this idea has been challenged recently [35]. In contrast, cytokinin *O*-glucosides were shown to be reversibly glycosylated and, because they are also resistant to cleavage by oxidases [77], they can act as CK storage forms. In our study, CK ribosides and ribotides were present at the highest concentrations over the experimental timeframe. tZ-*O*-glucosides gradually accumulated over time in all investigated tissues, suggesting that they might play an important role in CK homeostasis as cacao seedlings develop. In Arabidopsis, total *O*-glucosides were found to remain invariable as seedling developed [74], thus the increase in *O*-glycosylation in developing seedlings might be specific to cacao.

Overall, our study found parallel IAA and iP concentration gradients, and an inverse tZ pattern in relation to IAA and iP, as cacao root and hypocotyls developed (Figure 8), suggesting an inter-relation of these hormones during cacao seedling establishment. Crosstalk between auxin and cytokinin has been widely studied in Arabidopsis. In Arabidopsis roots, cytokinin signal transduction has been shown to modulate auxin levels by modulating its metabolism [27] and transport [78]. Genetic and molecular studies in Arabidopsis revealed that the maintenance of the root meristem is controlled by an AUX-PIN-SHY2-CKs regulatory module [79]. Indeed, *SHY2* (*SHORT HYPOCOTYL* 2) gene is activated by ARR1, a cytokinin signaling regulator, leading to SHY2-mediated downregulation of PIN auxin transporter proteins, and thus modulating auxin distribution. Conversely, auxin degrades SHY2 proteins resulting in PINs promotion and auxin redistribution. This interaction is centered on SHY2, which links the antagonistic action of auxins and CKs to maintain cell proliferation in the meristem while promoting cell type specification at the root transition zone. More recently, CKs were shown to control root meristem size by directly promoting the expression of the GH3.17 enzyme, which promotes auxin degradation and thus favors the transition from proliferation to cell elongation at the meristem [13]. Based on the hormonal dynamics captured in this study (Figure 8), we hypothesize that IAA, iP, and tZ are key hormonal players of the auxin-CKs crosstalk in developing cacao roots and hypocotyls. Lateral root organogenesis is also regulated by auxin-CKs crosstalk. Lateral root formation is a multistage process starting at the transition zone and culminating at the differentiation zone [80]. Bielach et al. [15] reported a repartition of IAA and CKs at the lateral root initiation site characterized by a peak of cytokinin between consecutive lateral roots, whereas IAA accumulated at the tip of newly formed lateral roots. Furthermore, CKs operate in lateral root organogenesis through PIN1-dependent modulation of cellular auxin distribution [81]. In our context, the reason for IAA/iP substantial increment in the Dz at day 10 could be attributed to a higher lateral root density observed in cacao seedlings at this stage. Lastly, hypocotyl elongation in Arabidopsis has been shown to be promoted by CKs [22]. Our study found tZ as the main active hormone that increased in concentration as hypocotyl elongated between 4 and 7 DAI, suggesting a role for tZ in the development of cacao hypocotyl during seedling establishment.

## 4. Materials and Methods

### 4.1. Plant Material and Growth Conditions

The present study was performed with the cacao genotype “PA 150” (PA stands for Parinari, a local name in Peru), which is included in the gene-bank of the Institute of Agricultural Research for Development at Nkolbisson (Yaounde, Cameroon). This genotype is known for its high productivity and its resistance to Phytophthora [82]. Fresh seeds were harvested from mature cacao pods. After removal of the seed coat, they were surface sterilized by immersion in 3% (*v/v*) sodium hypochlorite solution plus a few drops of Tween 20 for 20 min, and then rinsed three times in sterile water (10 min for each rinse). Seeds were sowed in jars containing half-strength DKW medium [83] under sterile conditions and incubated in a growth chamber for 10 days in darkness at 25 ± 1 °C.

### 4.2. Tissue Sample Collection

Germinated cacao seedlings were sampled at three different times: 4, 7, and 10 days after initiation of germination (DAI); a time frame that encompasses the development of lateral roots (Figure 1). Samples of interest were the hypocotyl segment (H) and four different developmental regions of the main root axis: meristematic zone (Mz), elongation zone (Ez), differentiation zone (Dz), containing emerging lateral roots, and mature differentiation zone (mDz), containing developed lateral roots. The different sections were macroscopically identified, measured, and collected as follows (Figure 1D). Mz was sampled by sectioning about 5 mm of the root tip. Ez was sampled 4 mm consecutively above the Mz. Dz was sampled by sectioning about 5 mm within the region where lateral root outgrowths were apparent. H was sampled by sectioning 5 mm of the region immediately below the fixation point of the cotyledons. At 7 and 10 DAI, lateral roots remarkably elongate just above the Dz and the corresponding region is referred to as mDz. A section of about 5 mm was sampled within the mDz. All samples were weighed, immediately plunged into liquid nitrogen, and stored at −80 °C. Three biological replicates were collected and each was a pool of three plants.

### 4.3. Quantitative Analysis of Auxins and Cytokinins

Simultaneous quantification of CKs and auxins was performed from the same samples by a modified method [84]. Briefly, after grounding the plant material in liquid nitrogen, the sample was mixed with 1 mL of cold extraction mixture of methanol/water/formic acid (modified Bieleski buffer—15/4/1, *v/v/v*). After adding isotopically-labeled internal standards (Olchemim Ltd., Czech Republic) together with 3 ceramic beads, the samples were homogenized using a MixerMill MM 301 bead mill (Retsch) at a frequency of 25 Hz for 12 min, followed by incubation for 10 min at 4 °C employing continuous shaking, then centrifuged for 15 min, 14,000 rpm at 4 °C. Supernatants were collected and reconstituted in 7 mL of 1 M formic acid prior to purification by solid-phase extraction (SPE) on MCX 1 cc/30 mg columns (Waters Inc.). MCXs were conditioned with methanol and water, equilibrated with 1 mL 50% (*v/v*) nitric acid, 2 mL of water, and 1 mL of 1 M formic acid. Then the sample was applied, the columns were washed with 1 mL of 1 M formic acid and eluted with the following order of solutions: 1 mL of methanol (to collect the IAA metabolites); 1 mL of 0.35 M ammonium hydroxide followed by 2 mL of 0.35 M ammonium hydroxide in 60% (*v/v*) methanol solution (CKs fraction). The collected eluates were vacuum-dried using a SpeedVac concentrator (Jouan, Winchester, UK), dissolved in 40 μL of 10% methanol and stored at −20 °C until mass spectrometry analysis. Quantification of compounds was performed using a 1290 Infinity LC system and 6490 Triple Quadrupole MS system (Agilent Technologies). IAA metabolites quantification was done according to Novák et al. 2012 [40], CKs quantification was carried out in accordance with Antoniadi et al. 2015 [37]. To determine the concentrations, MassHunter software (version B.05.02; Agilent Technologies) was used. For each independent sample, three technical replicates were performed.

### 4.4. Statistical Analysis

A one-way ANOVA with Welch’s correction for unequal variances followed by the Turkey’s post hoc were used to compare the results for all root and hypocotyl tissues. Analyses were applied to a 95% significance level. Statistical analysis was carried out using statistical software package R commander (R version 3.6.1).

## 5. Conclusions

We have undertaken the first detailed quantification of auxin and cytokinin metabolites in developmental root sections and hypocotyl of developing cacao seedlings. Our results highlight developmental gradients of IAA, iP and tZ associated to cacao root and hypocotyl growth during early seedling establishment. Moreover, we show that N-glycosylation of iP and O-glycosylation of tZ and DHZ are the most relevant metabolic pathways regulating the levels of active CKs during early cacao seedling development. Our study additionally found that IAA levels in developing cacao seedlings are predominantly regulated by the formation of amide-linked IAA catabolites and not by IAA oxidation, which provides the first evidence of this distinctive IAA metabolism in angiosperms.

## Data Availability

The data presented in this study are available in the article.

## Supplementary Materials

The following are available online at https://www.mdpi.com/article/10.3390/plants10050967/s1, Table S1: List of the metabolites analyzed in this study, Table S2: Levels of endogenous auxin metabolites (pmol g−1 FW) in the hypocotyl and developmental root sections of cacao seedlings harvested at 4, 7 and 10 DAI., Table S3: Levels of endogenous cytokinins (pmol/g fresh weight) in the hypocotyl and developmental root sections of cacao seedlings harvested at 4, 7, and 10 DAI.
